# Detecting, reporting, and analysis of priority diseases for routine public health surveillance in Liberia

**DOI:** 10.11604/pamj.supp.2017.27.1.12570

**Published:** 2017-05-28

**Authors:** Joseph Asamoah Frimpong, Meeyoung Mattie Park, Maame Pokuah Amo-Addae, Peter Adebayo Adewuyi, Thomas Knue Nagbe

**Affiliations:** 1 Liberia Field Epidemiology Training Program, Monrovia, Liberia; 2 Rollins School of Public Health, Emory University, Atlanta, USA; 3 Ministry of Health, Monrovia, Liberia

**Keywords:** Public health, surveillance system, Liberia

## Abstract

An essential component of a public health surveillance system is its ability to detect priority diseases which fall within the mandate of public health officials at all levels. Early detection, reporting and response to public health events help to reduce the burden of mortality and morbidity on communities. Analysis of reliable surveillance data provides relevant information which can enable implementation of timely and appropriate public health interventions. To ensure that a resilient system is in place, the World Health Organization (WHO) has provided guidelines for detection, reporting and response to public health events in the Integrated Disease Surveillance and Response (IDSR) strategy. This case study provides training on detection, reporting and analysis of priority diseases for routine public health surveillance in Liberia and highlights potential errors and challenges which can hinder effective surveillance. Table-top exercises and group discussion lead participants through a simulated verification and analyses of summary case reports in the role of the District Surveillance Officer. This case study is intended for public health training in a classroom setting and can be accomplished within 2 hours 30 minutes. The target audience include residents in Frontline Epidemiology Training Programs (FETP-Frontline), Field Epidemiology and Laboratory Training Programs (FELTPs), and others who are interested in this topic.

## How to use this case study

**General instructions:** ideally, 1 to 2 instructors facilitate the case study for 8 to 20 students in a classroom or conference room setting. The instructor should direct participants to read a paragraph out loud, going around the room to give each participant a chance to read. When the participant reads a question, the instructor directs all participants to answer or engage in discussions. The instructor may split the class to play different roles or take different sides in answering a question. As a result, participants learn from each other, not just from the instructors. Specific instructor’s notes are included with each question in the instructor’s version of this case study.

**Audience:** residents in Frontline Field Epidemiology Training Programs (FETP-Frontline), Field Epidemiology and Laboratory Training Programs (FELTPs), and others who are interested in this topic.

**Prerequisites:** before using this case study, case study participants should have received training in Integrated Disease Surveillance and Response protocols.

**Materials needed:** laptop with Microsoft Office applications, flipchart or white board with markers

**Level of training and associated public health activity:** basic – public health surveillance

**Time required:** approximately 2 ½ hours

**Language:** English

## Case study material

- Download the case study student guide (PDF - 2.11 MB)
- Request the case study facilitator guide
